# Long-Lasting Effects of Prenatal Ethanol Exposure on Fear Learning and Development of the Amygdala

**DOI:** 10.3389/fnbeh.2018.00200

**Published:** 2018-09-04

**Authors:** Olga O. Kozanian, David J. Rohac, Niusha Bavadian, Alex Corches, Edward Korzus, Kelly J. Huffman

**Affiliations:** ^1^Department of Psychology, University of California, Riverside, Riverside, CA, United States; ^2^Division of Biomedical Sciences, University of California, Riverside, Riverside, CA, United States; ^3^Interdepartmental Graduate Program in Neuroscience, University of California, Riverside, Riverside, CA, United States

**Keywords:** prenatal alcohol exposure, basolateral amygdala, basomedial amygdala, central nucleus, FASD, PrEE, PAE

## Abstract

Prenatal ethanol exposure (PrEE) produces developmental abnormalities in brain and behavior that often persist into adulthood. We have previously reported abnormal cortical gene expression, disorganized neural circuitry along with deficits in sensorimotor function and anxiety in our CD-1 murine model of fetal alcohol spectrum disorders, or FASD (El Shawa et al., 2013; Abbott et al., 2016). We have proposed that these phenotypes may underlie learning, memory, and behavioral deficits in humans with FASD. Here, we evaluate the impact of PrEE on fear memory learning, recall and amygdala development at two adult timepoints. PrEE alters learning and memory of aversive stimuli; specifically, PrEE mice, fear conditioned at postnatal day (P) 50, showed deficits in fear acquisition and memory retrieval when tested at P52 and later at P70–P72. Interestingly, this deficit in fear acquisition observed during young adulthood was not present when PrEE mice were conditioned later, at P80. These mice displayed similar levels of fear expression as controls when tested on fear memory recall. To test whether PrEE alters development of brain circuitry associated with fear conditioning and fear memory recall, we histologically examined subdivisions of the amygdala in PrEE and control mice and found long-term effects of PrEE on fear memory circuitry. Thus, results from this study will provide insight on the neurobiological and behavioral effects of PrEE and provide new information on developmental trajectories of brain dysfunction in people prenatally exposed to ethanol.

## Introduction

Maternal consumption of alcohol during pregnancy can cause deleterious effects on offspring brain development and behavior. Fetal alcohol spectrum disorders, or FASD, refer to a range of physical, cognitive, emotional, and neurobehavioral effects after *in utero* alcohol exposure. FAS, or fetal alcohol syndrome, represents the most severe end of the spectrum. Epidemiological studies report that approximately 1% of children in the U.S are born with FAS, and 2%–5% of children born with FASD (May et al., 2009, 2014). However, these percentages are likely grossly underestimated as at least 10% of women over the age of 18, and close to 20% of women between 35–44 years old reported alcohol consumption during pregnancy (CDC behavioral risk factor surveillance, 2011–2013; Tan et al., 2015). Despite warnings from the FASD research community and more recently, the CDC, alcohol consumption during pregnancy is the number one *preventable* cause of mental retardation in the United States (Abel and Sokol, 1986; Sampson et al., 1997).

Children with FASD endure alcohol-induced cognitive, emotional and behavioral impairments throughout development that often persist into adulthood. Cognitive deficits often include learning disabilities, poor judgment and reasoning, and problems with attention and memory. Emotional and behavioral dysregulation include symptoms of anxiety and depression, as well as aggressive-irritable and risk taking behaviors (Brown et al., 1998; Mattson and Riley, 1998; Riley and McGee, 2005; Greenbaum et al., 2009). Neuroimaging studies in people with FASD have consistently shown morphological alterations in brain development including reductions in cranial vault and brain size, volumetric abnormalities in the frontal, parietal, temporal lobes of the neocortex and cerebellum (Archibald et al., 2001; Sowell et al., 2001; Goodlett et al., 2005; Riley and McGee, 2005; McGee and Riley, 2006; Astley et al., 2009; de Zeeuw et al., 2012). Overall white matter hypoplasia, along with abnormal corpus callosum formation has also been described in those with FASD (Riley et al., 1995; Clark et al., 2000; Archibald et al., 2001; Sowell et al., 2001; Roussotte et al., 2012). Additionally, prenatal ethanol exposure, or PrEE, can induce significant reductions in basal ganglia, thalamic and hippocampal volume in affected human offspring (Coles et al., 2011; Nardelli et al., 2011; Roussotte et al., 2012; Treit et al., 2013).

Although much can be learned about the biology of FASD using human studies, animal models of FASD allow us to identify neural and developmental mechanisms underlying the spectrum disorder. FASD rodent models have PrEE-induced phenotypes that mirror the human condition. Specifically, PrEE murine offspring are born with reduced brain and body weights, and have abnormal neocortical development including ectopic neuronal connectivity (Miller and Dow-Edwards, 1988; El Shawa et al., 2013; Abbott et al., 2016, 2018). PrEE-induced changes in basal ganglia, thalamus, hippocampus and corpus callosum have also been documented in mice (Livy and Elberger, 2008; Abbott et al., 2016). Some of these structural malformations result from neuron loss in rodent neocortex, cerebellum and hippocampus, and others from abnormal genetic patterning (Goodlett et al., 1990; Ryabinin et al., 1995; Ikonomidou et al., 2000; Maier and West, 2001; Livy et al., 2003; El Shawa et al., 2013; Abbott et al., 2018).

Despite growing evidence of adverse brain and behavior outcomes stemming from PrEE, our understanding of how PrEE impacts complex systems is limited. Specifically, little is known about the effects of PrEE in brain structures and circuitry involved in social-emotional learning and regulation, such as the amygdala. Interestingly, it has been shown that social withdrawal, pathological shyness, explosive and inappropriate emotionality, and inability to form normal emotional attachments are related to alterations in the amygdala during development (Joseph, 1999; Munson et al., 2006; Karl and Herzog, 2007). Hence, investigating the long-term effects of PrEE on subdivisions of the amygdala, as well as amygdala-related behaviors, could provide us with insight into the neuropathological bases of FASD.

In this study, we examine the effects of PrEE on fear memory learning and recall, as well as amygdala development in early and late adulthood. Fear conditioning is a useful classical conditioning paradigm used to assess learning and memory (Fanselow, 1980). The amygdalar complex is a neuroanatomical structure that is involved in conditioned fear as it plays an essential role in the acquisition and consolidation of information about conditioned stimuli (CS), as well as the expression of CS fear (Cousens and Otto, 1998; Maren, 1999; Sacchetti et al., 1999). Here, we investigate the impact of PrEE on fear conditioning to aversive stimuli at two developmental time points, early (P50) and late (P80+) adulthood. We then examine whether PrEE significantly alters components of brain circuitry associated with emotional regulation, fear conditioning and fear memory recall. Specifically, we assess the long-term effects of PrEE on the gross anatomy and cell packing density of several amygdalar subdivisions, including the basolateral complex (BLA), basomedial nucleus (BMA) and central nucleus (CeA). By examining brain structures that regulate fear memory recall at different time points in adult PrEE mice, we were able to correlate the PrEE-induced neuroanatomical effects on the amygdala with learning and memory development.

## Materials and Methods

### FASD Mouse Model (PrEE)

All experimental procedures were approved by the University of California, Riverside Institutional Animal Care and Use Committee (IACUC). Eight to 10-week-old CD-1 female mice were paired with non-sibling males and singly housed for the entire gestational period once vaginal plug was confirmed (gestational day, GD, 0.5). An ethanol self-administration mouse model was used as described previously (El Shawa et al., 2013; Abbott et al., 2016, 2018). Briefly, dams were weight-matched, divided into ethanol-treated and control groups, and provided with *ad libitum* access to standard mouse chow and either a 25% EtOH in water solution (experimental), or an isocaloric maltodextrin solution (control). A series of dam measures were taken to validate our exposure model, including dam food and liquid intake, dam plasma osmolality and dam blood ethanol content. On the day of birth (P0), both male and female experimental and control mice were cross-fostered with ethanol-naïve dams. One pup from each litter was used for fear conditioning experiments and the rest were distributed for other ongoing experiments. At P20, both control and PrEE mice were weaned and raised to either P50 or P80+ with *ad libitum* access to standard mouse chow and water until commencement of behavioral experiments.

### Behavioral Assays

**Figure 1:**
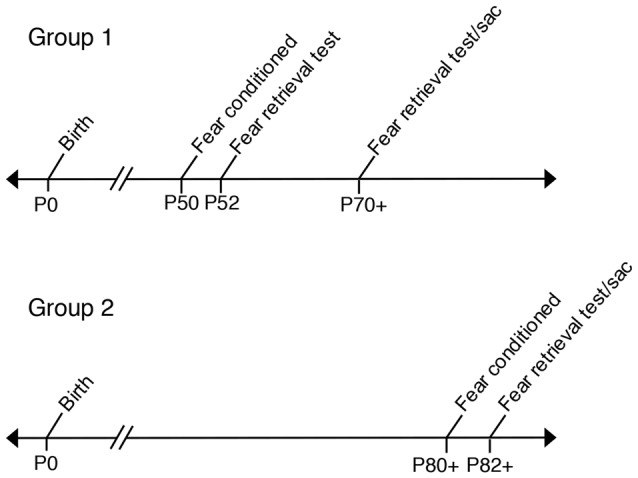
Timeline labeling age groups used in fear conditioning experiments. Group 1: Prenatal ethanol exposure (PrEE) and control mice were fear conditioned at P50 and tested for fear memory recall at P52. Same group of mice were retested for fear retrieval later in adulthood, at P70+ (P70–P72). Group 2: PrEE and control adult mice were fear conditioned at P80+ and tested on the fear retrieval task at P82+.

Two consecutive days prior to behavioral testing, all experimental and control offspring were brought to the experimental room, handled by trained experimenters and individually placed into training chambers for a 12-min acclimation period. Mice were fear conditioned in a standard sound attenuated chamber (context A) as previously described (Vieira et al., 2014). Initial 180 s of baseline activity was recorded to measure basal level of freezing, followed by four CS (tone, 75 dB and 2.8 kHz) paired with US (0.75 mA foot-shock. The US (foot-shock) was delivered amid the last 2 s of the 30 s CS duration. The US-CS pairings were separated by 180 s intervals. Animals that were fear conditioned at P50 were tested for tone fear retrieval on P52 in a sound-proof training chamber with new wall inserts (context B). One-hundred and eighty seconds of baseline activity was recorded to measure basal levels of freezing, followed by the onset of three tones (CS) with no foot-shock (US) in order to measure levels of freezing to each tone. Each tone fear retrieval trial was performed 180 s apart. All control and experimental animals fear conditioned at P50 and tested for fear retrieval at P52 were tested again for fear retrieval at P70+ (P70–P72). In a separate experiment, control and experimental mice underwent fear conditioning at P80+ and then, animals were tested for tone fear retrieval at P82+. All fear conditioning was performed in a computer-assisted fear-conditioning box (Coulbourn Instruments Inc., Holliston, MA, USA) located in a sound-attenuated chamber. Fear behavior testing was controlled by FreezeFrame (Actimetrics). Performance was scored by measuring freezing behavior, the complete absence of movement (Fanselow, 1980). Freezing was scored and analyzed automatically by a Video-based system (Freeze Frame software ActiMetrics Inc., Wilmette, IL, USA). Video was recorded at 30 frames/s. The Freeze Frame software calculated a difference between consecutive frames by comparing gray scale value for each pixel in frame. Freezing was defined based on experimenter observations and set as subthreshold activity for longer than 1 s. All freezing was expressed as “% freezing” and calculated as the percentage of time spent freezing. All tones used for training and tests were at the same frequency (2.8 kHz); see Figure 1 for behavioral analysis timeline.

### Brain and Body Weight Measures

Once behavioral experiments were completed, all mice used for fear conditioning and retrieval assays were weighed, euthanized via a lethal dose of sodium pentobarbital (100 mg/kg) and transcardially perfused with 0.9% saline followed by 4% paraformaldehyde in PBS (PFA, pH: 7.4). Brains were removed from the skulls and weighed at ages P70+ and P80+. A separate cohort of untrained PrEE and control mice were raised to P50, weighed, euthanized, perfused and brains collected and weighed for further anatomical analyses.

### Tissue Processing

After euthanasia and perfusion, brains were collected and cryoprotected. Tissue was cryosectioned at 30 μM in the coronal plane, mounted onto glass slides, stained for Nissl and imaged using a Zeiss Microscope and Zeiss Axiocamera.

### Neuroanatomical Measures

To test whether PrEE significantly affects subdivisions of the amygdala associated with fear conditioning and fear memory recall, we examined the basolateral amygdala (BLA) or basolateral complex, which includes the lateral, basal and accessory-basal nuclei, BMA, as well as the CeA in control and PrEE brains. Specific anatomical landmarks used to mark boundaries for BLA measurements included the amygdalar capsule, external capsule and boundaries of BMA and CeA, set by the external capsule and surrounding basic cell groups. Regions of interest (ROIs) in individual Nissl stained tissue sections were measured across all cases using an electronic micrometer using the ITCN plugin for ImageJ^1^ (Rasband, 1997) by a trained researcher blind to treatment group. ROIs were identified with the Allen Brain Mouse Atlas. Specific amygdala nuclei volumes were calculated by drawing borders around the BLA, BMA and CeA in serial sections at a fixed magnification (P50: 20×, P70+ and P80+:18×) using ImageJ. To determine whether altered amygdala nuclei volume observed in PrEE mice is generated by varied total cell number or degree of compaction, cell packing density within these ROIs was analyzed using the ITCN plugin for ImageJ.

### Statistical Analyses

The experimenters were blind to group conditions. N indicates sample size and error bars use the standard error of the mean. Statistical analysis was performed using Prism 7 (GraphPad). Two-way repeated measures analysis of variance (RM-ANOVA) with Bonferoni’s multiple comparisons were used to establish differences between control and PrEE fear conditioning training in both P50 and P80+ animals. Two-way ANOVAs followed by Bonferoni’s multiple comparisons were used to assess differences in fear retrieval tests. Two-sample independent *t*-tests were used to establish significant differences between body and brain weight and anatomical measures of all PrEE and control mice. For data displayed as a percent change, mean baseline corrected control was set as 100%, with experimental measures expressed as percentage variation from that mean. Significance values were set at *p* < 0.05.

## Results

### Dam Measures

As previously reported in our FASD mouse model (El Shawa et al., 2013), no significant differences were observed in dam food or liquid intake and blood plasma osmolality levels did not differ between experimental and control dams, ensuring our exposure paradigm did not cause malnutrition or dehydration (data not shown). Blood EtOH content (BEC) was measured in experimental dams at two gestational time points GD9 (BEC, 100–106 mg%) and GD19 (BEC, 130–140 mg%), an EtOH exposure model with BECs translationally relevant to and mirroring chronic alcohol abuse in humans (D’Souza El-Guindy et al., 2010).

### Behavioral Analyses

#### Auditory Fear Conditioning and Tone Fear Retrieval

**Figure 2:**
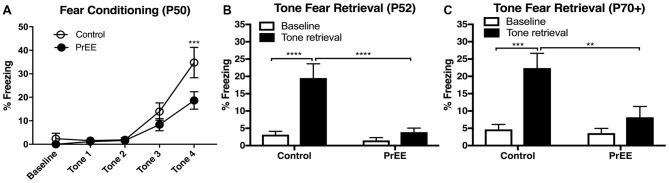
Fear conditioning and tone retrieval task in young adulthood. **(A)** Overall, PrEE mice showed significantly lower freezing behavior when trained on the fear-conditioning task during young adulthood (P50), as compared to controls (*P* < 0.05). **(B)** Contrary to PrEE mice, P52 control mice showed significantly higher levels of fear memory recall when tested on the tone fear retrieval task when compared to baseline levels (****P < 0.0001). PrEE mice showed significantly lower memory recall as displayed by freezing behavior when presented with the conditioned stimuli (CS), compared to control counterparts (****P < 0.0001). **(C)** Control mice maintained their fear memory recall at P70+ (P70–P72). When compared to their baseline levels (***P < 0.001). PrEE mice fear conditioned at P50 retained their fear memory, as there were significant differences between P70+ PrEE and control animals when presented with the CS during tone retrieval testing (**P < 0.01). P50 Control *N* = 18, PrEE *N* = 17; P70+ (P70–P72). Control *N* = 18, PrEE *N* = 17.

To determine whether our model produces deficits in learning and memory, we analyzed tone fear-conditioning and retrieval in PrEE and control adult mice at two developmental time points; P50 and P80+. PrEE mice exhibited lower freezing levels when conditioned for fear acquisition at P50, as compared to age matched controls (Figure 2A, *F*_(4,132)_ = 2.642, *P* < 0.05), which was evident during trial 4 (Figure 2A, *P* < 0.001). Data analyses from tone fear retrieval testing at P52 revealed significant differences in freezing to conditioned stimulus between PrEE and control mice (Figure 2B, two-way ANOVA of Group and Baseline/Retrieval Test, Group × Baseline/Retrieval interaction: *F*_(1,66)_ = 9.359, *P* = 0.0032, Baseline/Retrieval: *F*_(1,66)_ = 16.91, *P* = 0.0001, Group: *F*_(1,66)_ = 14.38, *P* = 0.0003). Young adult PrEE mice showed strong impairment in fear memory recall at P52, as indicated by notably lower freezing levels when compared to controls (Figure 2B, PrEE retrieval vs. Control retrieval, *P* < 0.0001). Both PrEE and Control groups showed the same baseline freezing (Figure 2B, *P* > 0.0001). In fact, PrEE mice show a similar level of fear response to baseline during tone fear retrieval at age P52 (Figure 2B, PrEE baseline vs. PrEE Retrieval, *P* > 0.9999). Additionally, mice fear conditioned at P50 and tested for fear recall 2 days later continued to show significantly impaired levels of fear memory recall in later adulthood, at P70–P72 (Figure 2C, two way ANOVA of Group and Baseline/Retrieval Test; Group × Baseline/Retrieval interaction: *F*_(1,66)_ = 5.345, *P* = 0.0239. Baseline/Retrieval: *F*_(1,66)_ = 15.42, *P* = 0.0002, Group: *F*_(1,66)_ = 7.237, *P* = 0.0090). While the Control group showed robust performance in cued fear memory retrieval in adulthood (Baseline-Ctrl vs. Retrieval-Ctrl, *P* < 0.002, Figure 2C), PrEE mice continue to display deficits in freezing responses to CS (Retrieval-Ctrl vs. Retrieval-PrEE, *P* < 0.0045), which did not differ from baseline performance (Baseline-PrEE vs. Retrieval-PrEE, *P* > 0.9999).

**Figure 3:**
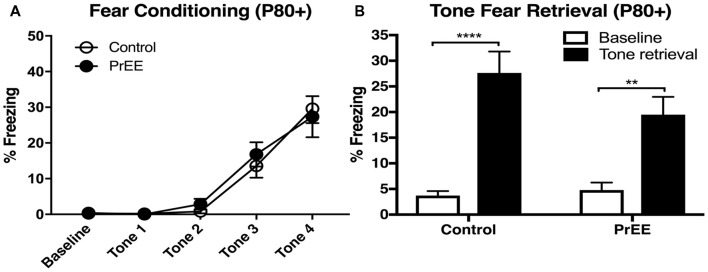
Fear conditioning and tone retrieval task in adult mice. **(A)** Adult control and PrEE mice showed no significant differences in freezing levels when trained on the fear-conditioning task (*P* > 0.05). **(B)** No significant differences in performance of control and PrEE mice during tone fear retrieval after 2-day delay (*P* > 0.05). Fear memory, however, was significantly higher in both control and PrEE animals, as shown in increased freezing behavior, when compared to their respective baseline levels (****P < 0.0001, **P < 0.01). P80+ Control *N* = 15, PrEE *N* = 14.

PrEE-related deficits observed in fear conditioning during young adulthood around P50, were not observed later on (Figure 3). When PrEE mice underwent cued fear conditioning training in later adulthood, around P80, they exhibit similar performance in fear acquisition (Figure 3A, *P* > 0.05) and tone fear retrieval (two way ANOVA of Group and Baseline/Retrieval Test; Group × Baseline/Retrieval interaction: *F*_(1,54)_ = 2.583, *P* = 0.1138; Baseline/Retrieval: *F*_(1,54)_ = 45.7, *P* < 0.0001; Group: *F*_(1,54)_ = 14.38, *P* = 1.527, *P* = 0.2220) when compared to Control animals. These data demonstrate a profound effect of prenatal alcohol abuse on maturation of fear-related behaviors during young adulthood.

#### Body and Brain Weights of PrEE and Control Mice

**Figure 4:**
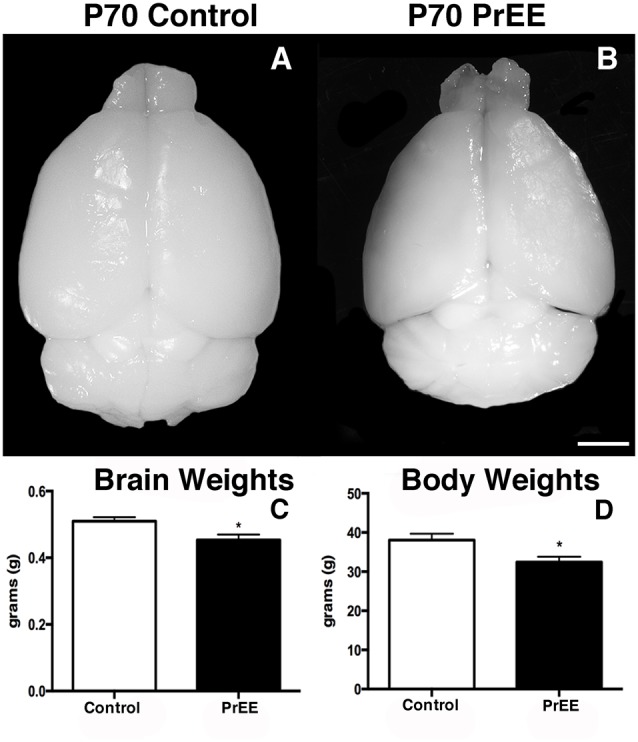
P70 brain and body weight measures. **(A,B)** Dorsal views of whole control and PrEE brains, respectively. PrEE brain size is reduced in size when compared to age-matched controls. **(C)** Average brain weights were significantly reduced in PrEE animals when compared to controls. **(D)** Body weights in PrEE animals were significantly lower compared to controls. *P < 0.05. Scale bar = 500 μm. P70–P72 Control *N* = 18, PrEE *N* = 17.

**Figure 5:**
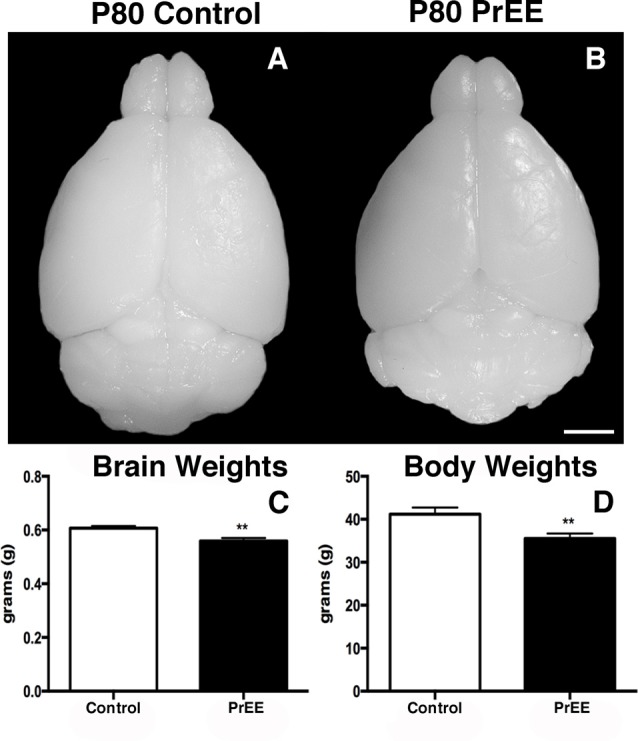
P80 brain and body weight measures. **(A,B)** Dorsal views of whole control and PrEE brains, respectively. **(C)** Brain weights in PrEE animals were significantly reduced compared to controls. **(D)** Average body weights were significantly lower in PrEE animals. **P < 0.01. Scale bar = 500 μm. P80+ Control *N* = 15, PrEE *N* = 14.

We have previously reported PrEE-induced reductions in brain and body weights in P0, P20 and P50 PrEE mice when compared to their control counterparts (El Shawa et al., 2013; Abbott et al., 2016). Interestingly, PrEE’s teratogenic effects on brain and body weight are maintained through later adulthood, in P70–P72 and P80+ mice. Visual assessment of brain size in P70–P72 control and PrEE animals (Figures 4A,B; respectively), as well as, P80+ control and PrEE mice (Figures 5A,B, respectively) revealed that *in utero* ethanol exposure can cause long-lasting reductions in brain size from birth to adulthood. Statistical analyses of brain weight measurements of PrEE and control brains confirmed the significant reductions in P70–P72 (Figure 4C, control 0.510 ± 0.012 and PrEE 0.453 ± 0.016, *P* < 0.05) and P80+ PrEE mice brains (Figure 5C, control 0.607 ± 0.008 and PrEE 0.560 ± 0.011, *P* < 0.01). Body weights were also significantly reduced in PrEE mice compared to controls at P70–P72 (Figure 4D, control 38.08 ± 1.600 and PrEE 32.44 ± 1.377, *P* < 0.05) and P80+ ages (Figure 5D, control 41.17 ± 1.544 and PrEE 35.52 ± 1.156, *P* < 0.01). These changes occur without altering brain/body weight ratios at any age (data not shown, *P* > 0.05).

### Neuroanatomical Analyses

#### Amygdalar Nuclei

**Figure 6:**
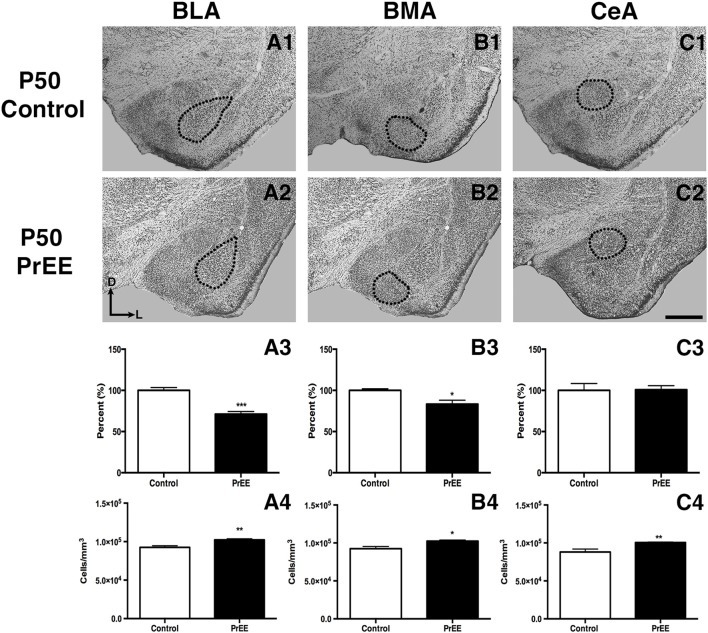
P50 amygdala size and cell packing density. Nissl stained coronal sections of control **(A1–C1)** and PrEE **(A2–C2)**. Outlines indicate area of measure. PrEE basolateral complex (BLA) was significantly smaller than controls (**A3**, ***P < 0.001), and had significantly higher cell packing density (**A4**, **P < 0.01). PrEE basomedial nucleus (BMA) was significantly smaller when compared to controls (**B3**, *P < 0.05), and had higher cell packing density (**B4**, *P < 0.05). There were no significant differences between control and PrEE CA size (**C3**, *P* > 0.05), however, PrEE CA had higher cell packing density (**C4**, *P* < 0.01). Scale bar = 500 μm. P50 Control *N* = 6, PrEE *N* = 6.

**Figure 7:**
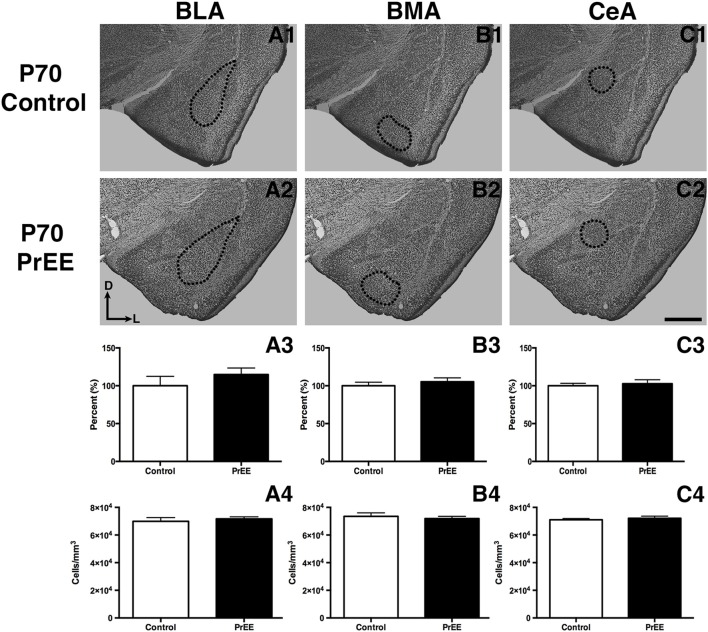
P70 amygdala size and cell packing density. Nissl stained coronal sections of control **(A1–C1)** and PrEE **(A2–C3)** brains. Outlines indicate area of measure. There were no significant differences between control and PrEE in all measures of amygdala nuclei volume (**A3–C3**, *P* > 0.05) and cell packing density (**A4–C4**, *P* > 0.05). Sections oriented dorsal (D) up and lateral (L) right. Scale bar = 500 μm. P70-P72 Control *N* = 7, PrEE *N* = 8.

**Figure 8:**
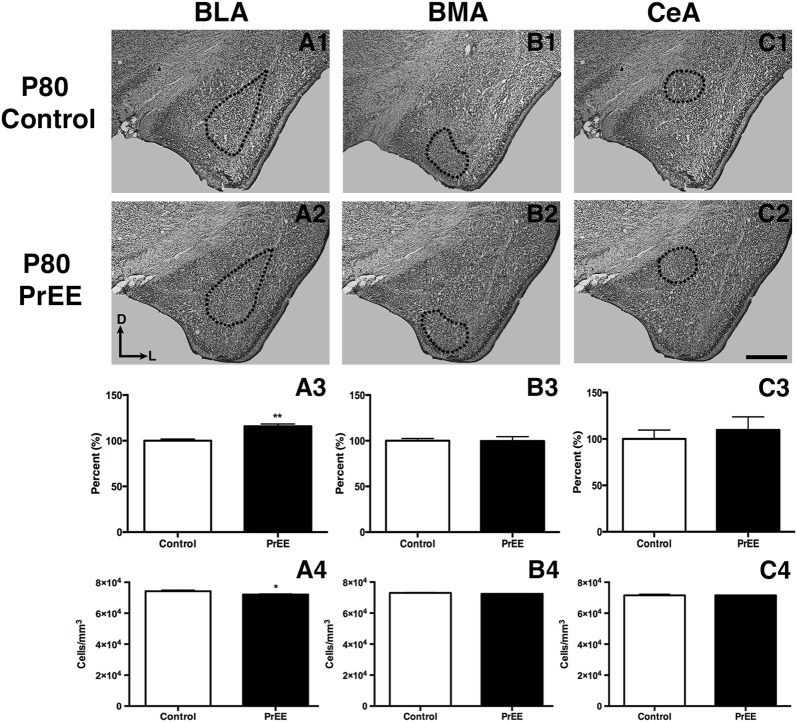
P80 amygdala size and cell packing density. Coronal sections of control **(A1–C1)** and PrEE **(A2–C2)** Nissl stains. Outlines indicate area of measure. PrEE BLA was significantly bigger than controls (**A3**, **P < 0.01) and had lower cell packing density (**A4**, *P < 0.05). There were no significant differences between control and PrEE BMA and CA size (**B3–C3**, *P* > 0.05) and cell packing density (**B4–C4**, *P* > 0.05). Sections oriented dorsal (D) up and lateral (L) right. Scale bar = 500 μm. P80+ Control *N* = 6, PrEE *N* = 7.

At P50, significant reductions were observed in PrEE BLA complex when compared to controls (Figures 6A1–A3, control 100 ± 3.391%, PrEE 71.21 ± 3.109%, *P* < 0.001). Similarly, the BMA of P50 PrEE mice were significantly reduced when compared to their control counterparts (Figures 6B1–B3, control 100 ± 1.907%, PrEE 83.31 ± 4.716%, *P* < 0.05). Conversely, there were no significant differences in CeA size between P50 PrEE and control animals (Figures 6C1–C3, *P* > 0.05). There were no significant differences in P70-P72 PrEE BLA, BMA and CeA as compared to control brains (Figures 7A1–A3,B1–B3,C1–C3, *P* > 0.05). P80+ BLA was significantly increased in mice exposed to ethanol *in utero* when compared to controls (Figures 8A1–A3, control 100 ± 1.760%, PrEE 115.8 ± 2.473%, *P* < 0.01). There were, however, no differences in BMA and CeA size between P80+ PrEE and control brains (Figures 8B1–B3,C1–C3, *P* > 0.05).

#### Amygdalar Nuclei Cell Packing Density

To determine the total cell packing density or varied cell number within each nuclei, we measured cell packing density in the BLA, BMA and CeA in P50, P70–P72 and P80+ animals. Cell packing density in P50 control and PrEE BLA, BMA and CeA was significantly different, with PrEE mice showing significant increases in all nuclei measures (Figure 6A4, BLA: control 92,544 ± 2,085, PrEE 1,02,261 ± 1,380, *P* < 0.01; Figure 6B4, BMA: control 92,435 ± 2,938, PrEE 1,02,500 ± 1,328, *P* < 0.05; Figure 6C4, CeA: control 88,073 ± 3,866, PrEE ± 1,00,528 ± 645.6, *P* < 0.01). There were no significant differences in P70–P72 BLA, BMA and CeA cell packing density between PrEE and control mice (Figures 7A4–C4, *P* > 0.05). Although analysis of P80+ BLA cell packing density showed significant decreases in PrEE brains (Figure 8A4, control 74,210 ± 759.6, PrEE 72,092 ± 430.3, *P* < 0.05), there were no differences between PrEE and control BMA and CeA (Figures 8B4,C4, *P* > 0.05).

## Discussion

### Fear Conditioning in Early Adulthood Disrupts Auditory Fear Memory Recall in PrEE Mice

We evaluated the impact of PrEE on fear conditioning and memory recall at two developmental time points. It is important to note that CD-1 mice generally display relatively low levels of baseline freezing in fear conditioning and fear memory testing tasks, a phenotype that is strain specific (Adams et al., 2002). Despite this, PrEE mice that were fear-conditioned at P50 showed significantly lower levels of fear learning and expression, compared to controls, when tested on fear memory recall 48 h post-conditioning and in later adulthood (P70–P72). Interestingly, PrEE mice fear-conditioned later in development (P80+) displayed similar levels of fear expression as controls when tested on fear memory recall. Results from our PrEE mice conditioned in late adulthood (P80+) are consistent with previous studies examining auditory fear memory recall in prenatal ethanol exposed adult rats between P130–P170. Specifically, Weeber et al. (2001) investigated fear recall following conditioning to an acoustic stimulus which resulted in the proper association of the tone (CS) with the footshock (US); they found no significant differences in freezing behavior in exposed to conditioned stimulus vs. control animals. Similarly, Schreiber and Hunt (2013) have also shown a recovery in fear conditioning and memory recall in adult P65–66 rats that were exposed to ethanol neonatally, indicating an age-related recovery of function. Conversely, in a more recent study, Brady et al. (2012) reported deficits in freezing to the CS (tone) in P90–P150 PrEE mice compared to controls. Here, the effect of *in utero* ethanol exposure on tone fear retrieval was examined in a novel context between groups that either experienced a fear conditioning training period or were untrained litter mates of control and PrEE mice. Although trained PrEE animals showed decreased freezing levels to the CS tone on test day, untrained PrEE and control animals exhibited equal levels of freezing, suggesting that the deficits seen in trained, exposed mice are dependent on the learning of the CS-US association (Brady et al., 2012). The opposing results described here may be due to differences in maternal ethanol exposure, as dams in the Brady study were exposed to ethanol both during gestation and a period of time prior to conception.

Despite PrEE animals showing similar freezing behavior as controls on test day when conditioned in late adulthood, PrEE mice showed deficits in freezing levels to the CS tone on test day when fear-conditioned earlier in development. It is possible that PrEE may delay the development of the brain circuitry or alter the physiology of areas and structures associated with fear conditioning and fear memory recall. In an elegant series of experiments, Hunt et al. (2009) have shown ethanol’s teratogenic effects on the developing brain in animals exposed to ethanol during the neonatal period and fear conditioned in adolescence (P30). They described a dose-dependent reduction in fear conditioning and memory recall, with the poorest performance observed in animals treated with the highest dose, as compared to their sham counterparts (Hunt et al., 2009). Therefore, it is possible that young adult mice exposed to ethanol *in utero* may need additional training trials to learn CS-US associations in order to perform at control levels when tested for tone fear retrieval.

### Effects of PrEE on Adult Body and Brain Weights

Previously, we reported reduced body and brain weights in newborn (P0), weanling (P20) and young adult (P50) PrEE mice (El Shawa et al., 2013; Abbott et al., 2016). Here, we extend those findings to later developmental ages (P70, P80). Although there were significant recoveries in amygdalar nuclei volume by P70 in PrEE mice, brain weight reductions related to PrEE continued through later stages of adulthood. This could be attributed to persistent PrEE-induced alterations in subcortical anatomy, neuronal loss in the cortex, cerebellum and hippocampus, as well as decreased white matter and glia in the brain (Bauer-Moffett and Altman, 1977; Goodlett et al., 1990; Ikonomidou et al., 2000; Abbott et al., 2016). PrEE mice also demonstrated lower body weights that persisted from birth to late adulthood (P80+) when compared to controls, although brain and body weight ratio remained unchanged following PrEE in all ages, suggesting that we are non-selectively inhibiting central nervous system development. Decreased body weight across development in our mouse model is consistent with outcomes resulting from maternal consumption of ethanol during pregnancy in humans and other rodent models of FASD (Margret et al., 2005; Chappell et al., 2007; May et al., 2014).

### PrEE Alters Amygdalar Gross Anatomy and Cell Packing Density

Certain aspects of amygdala development are vulnerable to the neurotoxic effects of PrEE. In our model, specific subdivisions of the amygdala, such as the basal lateral and medial nuclei, were dramatically reduced in volume and correlated with increased cell packing density from PrEE. These changes were correlated with altered fear learning, which is believed to result from activity within these limbic structures (Amano et al., 2011). Given that the amygdala is a key brain structure involved in emotional and social behavior, and socio-emotional abnormalities are common in humans with FASD, it is possible that abnormal amygdalar development could underlie some specific, socio-emotional and fear-response phenotypes observed in humans with FASD.

Early damage to the amygdala, regardless of etiology, has been shown to result in brain and behavioral dysfunction in humans and animal models. For example, lesions to the basolateral and central amygdala early in life resulted in a variety of adverse outcomes, including deficits in social interactions and stereotypic-like behaviors in adulthood (Wolterink et al., 2001; Gerrits et al., 2006). Amygdala volume reduction has also been correlated with symptoms of depression, anxiety, and autism, including impairments in social and communicative skills (Munson et al., 2006; Karl and Herzog, 2007). Similarly, studies assessing amygdalar volume and cell packing density in dementia and Alzheimer’s patients have reported shrinkage in overall amygdala size, with altered cell packing density, highlighting the role of volumetric measures and cell packing density in cognitive function and decline (Herzog and Kemper, 1980; Scott et al., 1992). Interestingly, many people with FASD experience the aforementioned disturbances (Streissguth et al., 1996); thus, it is plausible that the teratogenic effects of ethanol on the anatomy of the amygdala, as reported here, could have functional consequences leading to deficits in humans with FASD.

### Recovery of PrEE-Induced Amygdalar Phenotype in Later Adulthood: Delayed Development and Gene Expression

BMA and BLA are reduced in PrEE mice at P50, yet this effect appears to be mostly rescued by P70. One explanation is that ethanol may have induced a developmental delay in brain maturation, as has been suggested previously (Abbott et al., 2016). The phenotypes seen here, just beyond puberty, could result from PrEE’s teratogenic effects on amygdalar gene expression patterns, which could alter the trajectory of nuclei development. External insults early in development, such as environmental exposures and experiences like PrEE, have the ability to alter DNA methylation patterns, an epigenetic modification that plays an essential role in cell differentiation, gene imprinting, embryonic development and gene expression (Moore et al., 2013; Abbott et al., 2018). This epigenetic remodeling can, in turn, alter gene transcription and induce developmental abnormalities, as seen in animal models of FASD (Szyf, 2012, 2013a,b; El Shawa et al., 2013; Abbott et al., 2018).

Although DNA methylation has been thought to be a long-term and relatively stable epigenetic mark, developmental studies have revealed that this modification is not as static throughout development (Wu and Zhang, 2010). Therefore, it is possible that PrEE-induced changes in epigenetic markers and gene expression can alter the trajectory of physiological development in the limbic system and, in turn, delay the development of anatomical structures associated with fear conditioning and memory recall in PrEE.

### The Role of the Amygdala and Fear Learning

Although most consider the hippocampus as the primary brain structure involved in learning, the amygdala, with its complex connections within the limbic system and with the neocortex, plays an important role in learning general, and fear-related learning, specifically. For example, in tone fear conditioning, tone (CS)—foot shock (US) associations are directly encoded through synaptic plasticity in the amygdala, which receives direct auditory inputs (Medina et al., 2002). Amygdalar subdivisions have different functional properties in the circuitry involved in fear memory learning and conditioning. The lateral nucleus (LA) within the BLA is the main sensory input station of the amygdala for thalamic and cortical information about the CS, whereas the CeA is the output region that contributes most amygdala projections to brainstem fear effectors (Davis, 2000; LeDoux, 2000). There are, however, no direct connections between the main input (LA) and output (CeA) stations. Specifically, the LA nucleus projects to the CeA indirectly through the basal nuclei in the presence of interconnected clusters of GABAergic inhibitory neurons termed intercalated cell masses (ITCs) that are situated between the BLA (LA, BLA and BMA) and the CeA (McDonald and Augustine, 1993; Paré and Smith, 1993a,b). Because ITCs receive inputs from the BLA and project to the CeA, they play a crucial role in influencing the flow of information from the LA to the CeA (Collins and Paré, 1999). Thus, the basolateral and basomedial nuclei (BLA, BMA), serve to relay CS-related information from the LA to the CeA, bridging the gap between main sensory inputs to the amygdala and subsequent output to the brainstem (Amano et al., 2011). Studies examining the role of basal nuclei in fear conditioning circuitry have reported a reduction of conditioned freezing with combined BLA-BMA inactivation, highlighting the importance of basal amygdala nuclei in both the acquisition and expression of conditioned fear (Amano et al., 2011). Moreover, BMA neurons have been found to differentiate safe and aversive environments, with BMA activity suppressing fear-related freezing and elevated-anxiety states (Adhikari et al., 2015).

## Conclusion

Considering the brain circuitry involved in fear conditioning and memory, PrEE-induced anatomical alterations seen in the BLA, BMA and CeA earlier in adulthood may have functional consequences, leading to deficits in acquisition of aversive stimuli and subsequent fear expression in early and late adulthood. These PrEE-induced changes in the anatomy of the amygdala are correlated with abnormal fear conditioning during young adulthood, but also may have causal links. It is possible that the recovery seen in the BMA and CeA in older ages could account for the proper learning and expression of fear memory when conditioned later in adulthood, leading to comparable freezing behavior between PrEE and control animals when tested on fear memory recall. In conclusion, alcohol consumption during pregnancy can lead to abnormal development of key nuclei within the amygdala and, thus, learning deficits associated with amygdala dysfunction in the offspring. The results from this study demonstrate abnormal morphological development of the amygdala and related fear-conditioning and learning phenotypes in a mouse model of FASD. It is, however, important to note that although we observe a learning deficit in PrEE animals fear conditioned in young adulthood, further studies examining the effects of overtraining PrEE subjects in young adulthood and its result on the recovery of this deficit is warranted. This research helps elucidate possible underlying biological phenotypes that may lead to long-lasting behavioral deficits associated with FASD.

## Author Contributions

OK conducted experiments, analyzed data, created figures, wrote initial draft of manuscript. DR, AC and NB conducted experiments. EK helped design behavioral experiments and conducted initial behavioral analyses. KH designed experiments, supervised all data collection, analyses and figure creation, co-wrote manuscript with OK.

## Conflict of Interest Statement

The authors declare that the research was conducted in the absence of any commercial or financial relationships that could be construed as a potential conflict of interest.

## Footnotes

^1^https://imagej.nih.gov/
